# Effectiveness of a Goldilocks Work intervention in childcare workers – A cluster-randomized controlled trial

**DOI:** 10.5271/sjweh.4145

**Published:** 2024-04-01

**Authors:** Kathrine Greby Schmidt, Anders Fritz Lerche, Marie Raunkjær Christensen, Charlotte Lund Rasmussen, Leon Straker, Svend Erik Mathiassen, Andreas Holtermann

**Affiliations:** 1 The National Research Centre for the Working Environment, Copenhagen, Denmark.; 2 Department of Sports Science and Clinical Biomechanics, University of Southern Denmark, Odense, Denmark.; 3 School of Allied Health, Curtin University, Perth 6102, Australia.; 4 Centre for Musculoskeletal Research, Department of Occupational and Public Health Science, University of Gävle, Gävle, Sweden.

**Keywords:** high-intensity physical activity, RCT, workplace intervention

## Abstract

**Objective:**

Poor cardiorespiratory fitness and health is common among childcare workers. We designed the 'Goldilocks-games' according to the Goldilocks Work principle to provide high-intensity physical activity for childcare workers. We investigated the effectiveness of this Goldilocks Work intervention in increasing occupational high-intensity physical activity and improving work-related health.

**Methods:**

In a two-arm cluster randomized trial, 16 childcare institutions with 142 workers were randomly allocated to either an 8-week Goldilocks Work intervention or a control group. The primary outcome was occupational time in high-intensity physical activity. Secondary outcomes were occupational time in active physical behaviors, heart rate during sleep, pain, physical exhaustion, energy at work, work productivity, and need for recovery.

**Results:**

The intervention was successfully delivered and received. Both groups had a low amount of occupational high-intensity physical activity at baseline, and the intervention group reported playing the games 3.1 [standard deviation (SD) 1.5] times/week for a duration of 112.2 (SD 175.0) min/week. However, the intervention did not increase high-intensity physical activity or the secondary outcomes, except for energy at work, measured on a scale from 0–10, increasing 0.65 [95% confidence interval (CI) 0.08–1.21], and need for recovery, measured on a scale from 1–5, decreasing -0.32 (95% CI, -0.54– -0.09).

**Conclusion:**

The intervention was successfully delivered and received, but did not increase high-intensity physical activity. The intervention group increased their energy at work and decreased their need for recovery, but not the other health-related outcomes. Further research on how to design and implement health-promoting work environment interventions in childcare is needed.

With a projected increase of >50 000 children aged 0–5 years in Denmark by 2030 and an existing shortage of childcare workers (1), there is a great need to promote childcare workers work life expectancy. Since poor cardiorespiratory fitness and health issues are acknowledged barriers to a long and productive working life (2, 3), ensuring that childcare workers are healthy and fit is important. Beyond these demographic changes, prioritizing childcare workers’ well-being is paramount in its own right as a high proportion of childcare workers in Denmark suffer from musculoskeletal pain (38%), high sickness absence (19%) and poor cardiorespiratory fitness (16%) (4, 5), with likely consequences for their ability to adequately perform their job. Therefore, effective and sustainable interventions that improve childcare workers’ cardiorespiratory fitness and health are needed.

It is well documented that high-intensity physical activity can improve cardiorespiratory fitness and health (6). Thus, the low cardiorespiratory fitness and health among a high proportion of childcare workers could be a result of their physical activity being of insufficient intensity to increase cardiorespiratory fitness (7). Accordingly, we have found that childcare workers have only about 2.5 min of high-intensity occupational physical activity per day [≥60% of heart rate reserve (HRR)] (7). Therefore, high-intensity physical activity at regular intervals during work could be an effective strategy to improve their cardiorespiratory fitness and health. Workplace health promotion is, however, often arranged as an add-on, which requires time away from the productive work. As an alternative, the Goldilocks Work principle proposes to design productive work in a way that promotes fitness and health without compromising productivity (8, 9). This should be accomplished by re-designing productive work to offer a ‘just-right’ composition of occupational physical behaviors, for example by having a balance between high-intensity physical activity and recovery. Some studies testing the Goldilocks Work principle have shown generally positive results. One study (5) observed an increased duration of occupational high-intensity physical activity while another (10) found workers to be less fatigued, feel less pain and have more energy after an intervention according to the Goldilocks Work principle.

According to the European Act on Early Childhood Education and Care, an important pedagogical aim for childcare workers is to act as role models for promoting physical activity among the children (11). Based on the Goldilocks Work principle (9), we collaborated with childcare workers to develop pedagogical games (ie, 'Goldilocks-games') requiring the childcare workers to be physically active whilst acting as role models for the children. We introduced the Goldilocks-games to a panel of experts comprising members from childcare organizations, union representatives, representatives from employer associations, and occupational health consultants. This panel provided valuable insights and specific suggestions on how to tailor the games to align with the requirements, context, and work conditions in childcare settings. This included adapting the games to the new and improved educational curriculum (ie, six politically determined objectives) for childcare in Denmark (12). Subsequently, we tested the feasibility of the Goldilocks-games among childcare workers and found them to lead to substantial time in high-intensity physical activity compared to the most active period on a regular workday (5). Furthermore, the majority of the childcare workers considered the Goldilocks-games to be pedagogically relevant and usable as part of their daily work (5).

The aim of this cluster-randomized study was to investigate the effectiveness of an 8-week Goldilocks Work intervention implementing Goldilocks-games among childcare workers in increasing occupational high-intensity physical activity and improving work-related health (13). Thus, we assessed the effects on time at work in high-intensity physical activity (primary outcome) as well as (secondary outcomes) heart rate during sleep, time in active physical behaviors, pain, physical exhaustion, energy at work, need for recovery and work productivity. We also investigated the implementation of the intervention.

## Method

The protocol of the intervention has already been reported (13). The Danish National Committee on Biomedical Research Ethics (the local ethical committee of Frederiksberg and Copenhagen) has evaluated a description of the study and concluded that, according to Danish law as defined in Committee Act § 2 and § 1, the intervention described should not be further reported to the local ethics committee (reference number: H-18041423).

The COVID-19 pandemic required us to modify the intervention to match restrictions issued by the Danish Health Authority and requirements from the workplaces. We updated the modifications of the intervention in the trial record (ISRCTN15644757). To be transparent about the modifications, we have included a table in the supplementary material (www.sjweh.fi/article/4145) that outlines the specific modifications made.

We conducted the study as a two-arm cluster-randomized controlled trial with a wait-list control group receiving usual practice. The usual practice consisted of (i) ergonomics consultancy and guidance, (ii) individual advice on pain management from a physiotherapist and (iii) an occupational therapist employed in the local municipality. The intervention period was 8 weeks (supplementary appendix A, modification 1).

Our primary outcome is change in relative work time spent in high-intensity physical activity, according to heart rate data. Our secondary outcomes include assessing changes in heart rate during sleep, physical behaviors measured by accelerometry, and pain, physical exhaustion, energy at work, need for recovery and work productivity, all measured by questionnaires. Additionally, we conducted a process evaluation of the intervention, based on questionnaire answers from the childcare workers.

### Participants

We recruited childcare institutions (clusters) with assistance from the Work Environment Consultancy (WEC) of Copenhagen Municipality and via social media. In Denmark, childcare is divided into nursery for children age 0–3 years, focusing on basic development, and kindergarten for children age 3–6 years, introducing structured learning alongside play. Inclusion criteria in the present study were that institutions should (i) provide childcare for children aged 3–6 years (ie, kindergartens), and (ii) employ a minimum of nine childcare workers. All childcare workers at participating childcare institutions were eligible to participate. Exclusion criterion among childcare workers was expected termination of employment at the childcare institution during the intervention period. Written informed consent was collected for all participants.

### Blinding and randomization

Blinding of childcare workers was not possible. Before recruitment of childcare institutions, an independent researcher created a digital allocation sequence using the statistical software RStudio. The randomization of childcare institutions followed the digital allocation sequence. As soon as an institution agreed to participate, the independent researcher informed whether the institution was allocated to the intervention or control group.

### Procedures

Prior to the intervention, we developed (supplementary appendix A, modification 2) four additional Goldilocks-games similar to the Goldilocks-games previously co-developed with childcare workers, which were confirmed to provide occupational time in high-intensity physical activity (5). Thus, a total of seven Goldilocks-games were used in the intervention (14).

**Figure 1:**
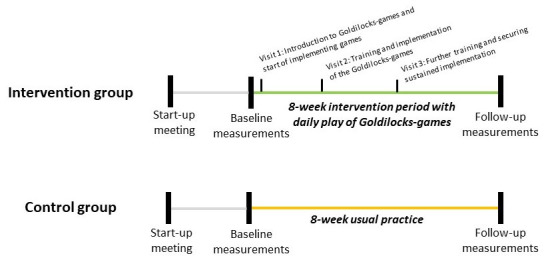
Timeline of activities in the intervention and control groups.

The activities in both intervention and control groups (figure 1) began with a start-up meeting between the manager, a union representative, and an occupational health and safety representative (collectively referred to as the TRIO) from the institution, a member of our research group, and the consultant from WEC who subsequently delivered the intervention. During the start-up meeting, the TRIO was introduced to the Goldilocks Work principle and the intervention contents. Additionally, a text message was sent to the private mobile phone of all eligible childcare workers before the intervention, containing a link to a video (supplementary appendix A, modification 3) explaining the intervention and project.

The main component of the intervention was to play the Goldilocks-games on a daily basis. To integrate the games in an already existing daily routine, the childcare workers were encouraged to play the games each time they went outside with the children. The consultant visited the institutions three times during the intervention period to educate the childcare workers and children in the Goldilocks-games and support their implementation and maintenance. On the first visit (supplementary appendix A, modification 4) (figure 1), the consultant spent half-an-hour with each group (ie, 18–20 children aged between 3–6 years and 2–3 childcare workers), teaching them two Goldilocks-games. To motivate the children, paw-print stickers and a poster showing an 8-week table (supplementary appendix B) were given to each group. The children were encouraged to place a sticker on the poster each time they had played a Goldilocks game. Two additional half-an-hour visits were conducted with each group in week three (supplementary appendix A, modification 5) and week five (supplementary appendix A, modification 6) (figure 1). The aim of these visits was to teach the remaining Goldilocks-games and reinforce the groups’ commitment to playing them.

### Data collection

Data was collected between April 2021 and April 2022. In agreement with the manager of each institution, we or the childcare workers themselves (supplementary appendix A, modification 7) conducted the baseline and follow-up measurements at the workplace during worktime. The baseline measurements were carried out one week prior to the beginning of the intervention period, whereas the follow-up measurements were conducted during the final week of the intervention period. If the childcare workers did the measurements themselves (supplementary appendix, modification 7), they used a manual for conducting anthropometric measurements and applying the heart rate and activity monitors.

### Anthropometric measurements

We or the childcare workers performed anthropometric measurements of height, weight, body mass index, fat percentage and resting blood pressure. Anthropometric measures were not collected on workers that (i) had fever on the test day, (ii) were pregnant, or (iii) had a pacemaker.

### Technical measurements

We or the childcare workers collected heart rate using a FirstBeat Bodyguard 2 heart rate monitor (FirstBeat Technologies Ltd., Jyväskylä, Finland). Childcare workers were asked to wear the heart rate monitor for five consecutive working days. Subsequently, we downloaded the heart rate data using the FirstBeat Uploader software. Beat error is a common phenomenon in heart rate measurements and refers to periods of missing heart rate data. A typical reason for beat error is bad electrode contact, which becomes more likely when the person is moving. A custom-made MatLab-based software (Acti4) was used to determine and later categorize the relative heart rate. Relative heart rate was expressed as percentage HRR, ie, the interval between the estimated maximal heart rate (15) and the resting heart rate. Resting heart rate was defined as the lowest recorded heart rate value during the first night’s sleep (16, 17). High-intensity physical activity during work was defined to occur at ≥60% HRR, as intensities above this threshold have been suggested to effectively improve cardiorespiratory fitness (18, 19). Workdays were considered valid if they contained ≥4 hours of measurements and ≤50% beat error, in line with previous studies using heart rate monitors (16, 20, 21).

While not mentioned in the original protocol, accelerometer measurements were included to provide additional information on physical activity as a secondary outcome. An accelerometer (3-Axis Logging Accelerometer; Axivity Ltd, Newcastle upon Tyne, UK) was attached to the right thigh with adhesive tape at the most prominent part of the quadriceps femoris muscle, and secured with transparent adhesive film. The software OMGUI was used to initiate accelerometers and download accelerometer data. Workdays were considered valid if they contained ≥4 hours of accelerometer measurements (7, 21). We did not collect heart rate or accelerometer measurements on childcare workers that (i) were allergic to adhesive tape, (ii) had fever on the test day or (iii) were pregnant. During the five test days, the childcare workers were instructed to note in a diary what time they (i) got out of bed, (ii) arrived at work, (iii) left work, (iv) went to sleep, (v) if any of the devices were detached, and (vi) any days when they were absent from work due to sickness or leave. The diary was digitized and used to categorize heart rate and accelerometer measurements into ‘working hours’, ‘leisure time’, and ‘sleep’ (22). The accelerometer measurements during working hours were classified into two categories, ie: (i) non-active physical behaviors (lie and sit) and (ii) active physical behaviors (standing, moving, walking, running, stair climbing and bicycling).

### Questionnaires

A week before baseline and at the 8-week follow-up, the childcare workers received a text message containing a unique link to a digital questionnaire. The questionnaire consisted of questions regarding (i) sociodemographic factors, (ii) health behaviors, and iii) questions on secondary outcomes, ie, pain ("How much pain do you experience in your body during the work day?"), physical exhaustion ("How physically tired are you during the work day?") and energy at work ("How much energy do you have during the work day?"). All three questions were measured on 11-point (0–10) Likert scale. Work productivity was measured using a single question ("How would you assess your productivity? How much have you accomplished in your work over the past month?") on a 11-point (0–10) Likert scale (23). Need for recovery was evaluated with three questions ("At the end of my work day I am exhausted", "I find it hard to show interest in other people when I have just come home from work" and "It takes me over an hour before I am fully recovered/fully improved after a work day") scored on scales from 1–5 with response categories: never, rarely, sometimes, generally, and always (24). For the analysis, a composite score was developed by calculating the mean of the three answers (25, 26).

### Process measures

Childcare worker attendance at the three visits was registered. In the 8-week follow-up survey, the childcare workers were asked how often and for how long they participated in playing the Goldilocks-games. Moreover, they were asked questions about their appraisal of the intervention including their desire to continue, level of satisfaction, and if they would recommend the program to other childcare institutions.

### Statistical analyses

Anthropometrics and process data variables were summarized with group mean and standard deviation (SD) across all clusters, ie, daycare institutions.

The intervention effect on the primary outcome, ie, relative occupational time in high-intensity physical activity, was evaluated following a compositional data analysis (CoDA) approach (27). Each childcare worker’s average daily occupational time at baseline and at follow-up was described as a composition consisting of occupational time with HRR≥60% and occupational time with HR<60%. This occupational time composition was transformed to an isometric log-ratio (ilr_1_), calculated as:


$$ilr₁=\sqrt{\frac{1}{2}}\ln\left(\frac{Occupational\;time\;with\;HRR\geq 60\%}{Occupational\;time\;with\;HRR<60\%}\right)$$

The same method was applied to worker’s average daily occupational time in active physical behaviors to produce an ilr expressing active relative to non-active physical behaviors (ilr_2_). For the primary and secondary outcomes the ilrs were analysed as an outcome variable.

The effect of the intervention on the primary and secondary outcomes were evaluated using mixed models. In all models, childcare institution was entered as random cluster effect. Fixed effects were point-in-time (baseline and follow-up), group (intervention and control) and an interaction term between point-in-time and group. Conclusions about the effectiveness of the intervention were based on the interaction effect between point-in-time and group, and we set the statistical significance at P<0.05 for a 2-sided test.

All analyses were performed in R version 4.1.3, using the compositions and lme4 packages (28, 29).

## Results

### Demographics of participants

**Figure 2:**
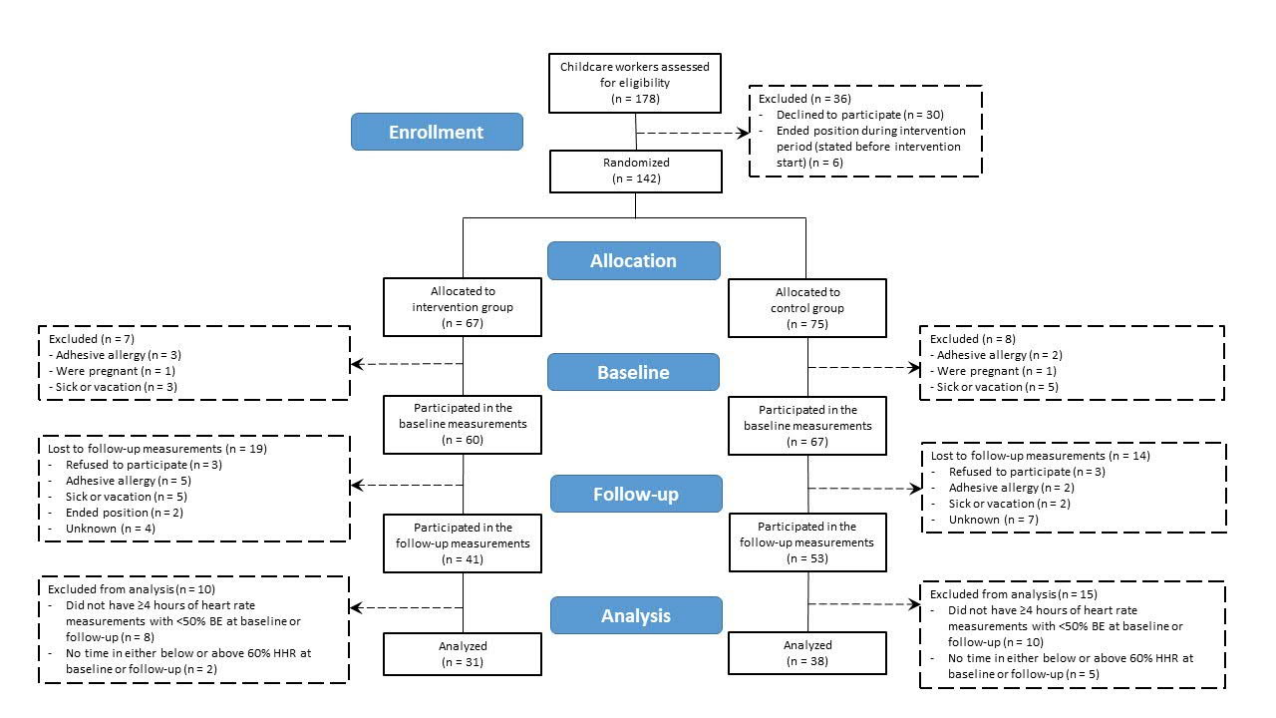
CONSORT flow diagram for heart rate measurements (primary outcome).

A total of 16 institutions were allocated to the intervention (N=7) and control (N=9) groups. Out of 178 eligible childcare workers, 142 were enrolled in the study (figure 2). Table 1 shows the demographics of the total sample and of workers included in the statistical analysis of the primary outcome, separated into intervention and control groups. The demographic profiles of the intervention and control groups were similar to the total sample. Most were female (intervention 74.2%; control 71.1%), born in Denmark (intervention 90.3%; control 84.2%) and approximately one quarter were smokers (intervention 29.0%; control 26.3%). Mean age was 43.4 (SD 11.3) and 39.6 (SD 13.4) years in the intervention and control group, and about half the childcare workers were categorized as overweight/obese (intervention 54.8%; control 44.7%).

**Table 1:** Baseline characteristics of the study population: Total sample versus childcare workers included in the analysis of the primary outcome, separated into the intervention and control group. [SD=standard deviation; BMI=body mass index.]

		Total (N = 142)			Intervention (N = 31)			Control (N = 38)	
		N (%)	Mean (SD)		N (%)	Mean (SD)		N (%)	Mean (SD)
Gender									
	Female	99 (69.7)			23 (74.2)			27 (71.1)	
	Male	43 (30.3)			8 (25.8)			11 (28.9)	
	Missing	0 (0.0)			0 (0.0)			0 (0.0)	
Age (years)			40.9 (12.6)			43.4 (11.3)			39.6 (13.4)
	Missing	0 (0.0)			0 (0.0)			0 (0.0)	
Ethnicity									
	Born in Denmark	122 (85.9)			28 (90.3)			32 (84.2)	
	Born outside Denmark	14 (9.9)			3 (9.7)			4 (10.5)	
	Missing	6 (4.2)			0 (0.0)			2 (5.3)	
Job title									
	Childcare worker	80 (56.3)			20 (64.5)			22 (55.3)	
	Childcare assistant	47 (33.1)			10 (32.3)			14 (36.8)	
	Other	8 (5.6)			1 (3.2)			1 (2.6)	
	Missing	7 (5.0)			0 (0.0)			1 (5.3)	
Seniority at current workplace									
	<3 months	12 (8.5)			2 (6.5)			6 (15.8)	
	3 months– <1 year	25 (17.6)			4 (12.9)			8 (21.1)	
	1 year– <3 years	28 (19.7)			7 (22.6)			5 (13.2)	
	3 years– <5 years	16 (11.3)			4 (12.9)			2 (5.3)	
	5 years– <10 years	25 (17.6)			7 (22.6)			10 (26.3)	
	≥10 years	29 (20.4)			7 (22.6)			5 (13.2)	
	Missing	7 (4.9)			0 (0.0)			2 (5.3)	
Self-reported time in main occupation (hours/week)			34.3 (4.1)			35.0 (3.3)			34.4 (4.3)
	Missing	8 (5.6)			0 (0.0)			2 (5.3)	
Smoking									
	Yes	33 (23.2)			9 (29.0)			10 (26.3)	
	No	99 (69.7)			22 (71.0)			26 (68.4)	
	Missing	10 (7.1)			0 (0.0)			2 (5.3)	
BMI (kg/m^2^)									
	Underweight (<18 kg/m^2^)	2 (1.4)			0 (0.0)			0 (0.0)	
	Normal weight (18–25 kg/m^2^)	68 (47.9)			14 (45.2)			21 (55.3)	
	Overweight/obese (>25 kg/m^2^)	69 (48.6)			17 (54.8)			17 (44.7)	
	Missing	3 (2.1)			0 (0.0)			0 (0.0)	
	Blood pressure (mmHg)								
	Normal (≤130 and ≤80 mmHg)	75 (52.8)			18 (58.1)			18 (47.4)	
	Elevated (130–>140 and 80->90 mmHg)	36 (25.4)			6 (19.4)			11 (28.9)	
	Hypertension (≥140 or ≥90 mmHg)	29 (20.4)			7 (22.6)			9 (23.7)	
	Missing	2 (1.4)			0 (0.0)			0 (0.0)	

### Measurements

*Heart rate.* Of the 142 childcare workers enrolled in the study, 127 completed the heart rate measurements at baseline, the remaining 15 (intervention, N=7; control, N=8) either declined or were unable to participate. Among the 127 childcare workers who completed the heart rate measurements at baseline, 94 (intervention, N=41; control, N=53) completed the measurements at follow-up, the remaining 33 were lost to follow-up (intervention, N=19; control, N=14). Among the 94 childcare workers who completed the heart rate measurements at follow-up, 25 (intervention = 10; control, N=15) were excluded from the analysis due to not meeting the quality criteria. Data from the remaining 69 (intervention, N=31; control, N=38) fulfilled the quality criteria and were included in the statistical analysis of heart rate measurements.

*Accelerometry.* Out of the 142 childcare workers included in the study, 130 (intervention, N=62; control, N=68) agreed to participate in the accelerometer measurements of physical behaviors at baseline. Of these, 100 completed the measurements at follow-up. Two childcare workers were excluded as their data did not meet the quality criteria. Data form the remaining 98 childcare workers’ data (intervention, N=42; control, N=56) fulfilled the quality criteria and were included in the statistical analysis of physical behaviors.

*Questionnaires*. The secondary outcomes measured using questionnaires included 128 childcare workers at baseline (intervention, N=61; control, N=67) and 109 childcare workers at follow-up (intervention, N=49; control, N=60). In total, 104 childcare workers fulfilled the quality criteria and were included in the statistical analysis for physical exhaustion and energy at work (intervention, N=46; control, N=58), 103 were included for pain and work productivity (intervention, N=45; control, N=58), and 102 for need for recovery (intervention, N=44; control, N=58). In total, 71 childcare workers (intervention, N=31; control, N=40) fulfilled the quality criteria and were included in the statistical analysis of sleeping resting heart rate.

### Effects of the intervention

Table 2 shows the intervention effects on the primary and secondary outcomes. Intention-to-treat analysis showed no statistically significant intervention effect on occupational time in high-intensity physical activity (-0.36, 95% CI -1.10–0.37), nor on heart rate during sleep, occupational time in active physical behaviors, pain, physical exhaustion or work productivity following the 8-weeks intervention. However, there was a statistically significant increase in self-reported energy at work, measured on a scale from 0–10, of 0.65 (95% CI 0.08–1.21) and a decrease in need for recovery, measured on a scale from 1–5, of -0.32 (95% CI -0.54– -0.09) in the intervention compared to control group.

**Table 2:** Intervention effects on primary and secondary outcomes. [CI=confidence interval; HRR=heart rate reserve; IIr=isometric log-ratio; SD=standard deviation.]

Variable		Time		Intervention group			Control group			Estimated treatment effect		P-value
				N	Mean (SD)		N	Mean (SD)		Mean	95% CI	
Primary outcome												
	Heart rate (hours/day)											
		<60% HRR	Baseline	31	6.08 (0.89)		38	6.49 (1.10)				
			Follow-up	31	6.17 (1.02)		38	6.31 (0.85)				
		≥60% HRR	Baseline	31	0.08 (0.15)		38	0.02 (0.04)				
			Follow-up	31	0.07 (0.13)		38	0.03 (0.05)				
Ilr_1_ ≥60% HRR vs. <60% HRR										-0.36	-1.10–0.37	0.34
Secondary outcomes												
	HR during sleep (beats/minute)											
			Baseline	33	48.2 (7.2)		44	48.0 (6.2)				
			Follow-up	33	47.6 (8.3)		44	47.9 (6.8)		0.51	-1.51–2.55	0.63
	Physical behaviors (hours/day)											
		Non active	Baseline	42	5.37 (0.75)		56	5.70 (0.65)				
			Follow-up	42	5.41 (0.84)		56	5.54 (0.63)				
		Active	Baseline	42	1.11 (0.30)		56	1.23 (0.36)				
			Follow-up	42	1.20 (0.31)		56	1.28 (0.38)				
Ilr_2_, active vs. non active										0.01	-0.05–0.06	0.25
	Pain (0–10)											
			Baseline	45	3.7 (3.0)		58	2.7 (2.3)				
			Follow-up	45	3.6 (2.9)		58	3.0 (2.3)		-0.28	-1.02–0.46	0.47
	Physical exhaustion (0–10)											
			Baseline	46	3.9 (2.3)		58	4.1 (2.0)				
			Follow-up	46	3.5 (2.4)		58	3.9 (2.2)		-0.17	-0.97–0.65	0.69
	Energy at work (0–10)											
			Baseline	46	6.9 (1.7)		58	7.5 (1.3)				
			Follow-up	46	7.4 (1.6)		58	7.2 (1.5)		0.65	0.08–1.21	0.03
	Need for recovery (1–5)											
			Baseline	44	3.3 (0.8)		58	3.1 (0.7)				
			Follow-up	44	3.2 (0.9)		58	3.3 (0.8)		-0.32	-0.54– -0.09	0.01
	Work productivity (0–10)											
			Baseline	45	7.3 (1.5)		58	7.3 (1.4)				
			Follow-up	45	7.7 (1.3)		58	7.3 (1.3)		0.27	-0.31–0.85	0.36

### Delivery and adherence to intervention

As planned, one start-up meeting was held in each of the 16 childcare institutions enrolled in the study (100%) (table 3). In total, 26 of those 31 childcare workers in the intervention group, who were included in the statistical analysis of the primary outcome, answered the questions regarding Goldilocks-games. Of these, 80.6%, 77.4% and 67.7% participated in the first, second and third visit, respectively. Twelve (38.7%) childcare workers participated in all three visits, fifteen (47.8%) participated in two visits, and four (14.9%) participated in only one visit (data not shown). On average, the childcare workers played Goldilocks-games 3.1 (SD 1.5) times/week for an average duration of 112.2 (SD 175) minutes/week.

**Table 3:** Participation in visits and time played Goldilocks-games.

Intervention activities		Dose delivered			Dose received			
		Planned (N)	Delivered (%)		N (%)	Mean	Percentile	
							25^th^	75^th^
Start-up meeting		16	100		N/A			
Visits								
	Visit 1	21	100		25 (80.6)			
	Visit 2	21	100		24 (77.4)			
	Visit 3	21	100		21 (67.7)			
Goldilocks-games (N=26)								
	Time/week					3.1	2	4
	Minutes/week					112.2	30	120

### Appraisal of intervention

Most childcare workers were satisfied with the intervention to a high/very high extent (66.0%) and the majority wanted to continue with the Goldilocks-games either partly (42.6%) or to a high/very high extent (46.8%). In addition, a large proportion considered the intervention to be relevant for other childcare institutions to a high/very high extent (76.6%). Furthermore, a majority of the childcare workers reported that COVID-19 restrictions to none/low extent made it difficult to complete the visits (80.8%) and the Goldilocks-games (89.4%) (data not presented).

## Discussion

The intervention was feasible to conduct and deliver, and well-received by the childcare workers. We found no significant effect on occupational time in high-intensity physical activity and the secondary outcomes, except for a significant increase in self-reported energy at work and a decrease in need for recovery in the intervention group compared with the control group.

The main finding of our intervention was the absence of a significant effect on occupational time in high-intensity physical activity. This may have been due to measurement limitations or implementation failure. An important measurement limitation was that valid heart rate measurements were only available for 69 of a total of 127 childcare workers (intervention: N=31, control: N=38). This considerable exclusion of data (either due to insufficient measurement duration or low data quality) may have resulted in the heart rate measurements not providing a valid representation of occupational time in high-intensity physical activity. However, the accelerometer data on occupational physical behavior did not show a significant intervention effect either.

Therefore, the lack of intervention effect on the primary outcome is more likely to be explained by implementation failure in terms of how the intervention was delivered, received and implemented. We successfully conducted a start-up meeting in each childcare institution, ensuring that the institutions received adequate information regarding the intervention. The visits, aimed at educating the childcare workers in the Goldilocks-games, were conducted with good participation rates. Thus, the childcare workers likely had the necessary competence to carry out the intervention as intended.

Childcare workers were encouraged to play Goldilocks-games when they went outside with the children, as daily outside activities are routine in Danish childcare. Despite the challenges posed by the pandemic, the childcare workers reported playing the games 3.1 times per week, on average, for a total of 112.2 minutes per week. Therefore, although the frequency and duration of implementation was somewhat less than intended, the duration fulfilled the minimum amount of time in high-intensity physical activity to improve fitness. Thus, the lack of an intervention effect is likely not to be explained by the childcare workers not playing Goldilocks-games sufficiently often or for sufficient duration.

As the childcare workers received the information needed to carry out the intervention as planned, and reported to play the Goldilocks-games several times per week, a more likely implementation failure relates to the extent to which they adhered to the Goldilocks-games protocol, stating that the games should be played intensely. In a pilot study, we documented that the games can provide more minutes (18/33 minutes) in high-intensity physical activity than the most active period of equal length (0.5/33 minutes) on a regular workday (5). However, it appears that the childcare workers in the current study conducted the games in a way that did not require them to be physically active to a sufficient extent. This hypothesis is supported by the absence of significant changes in occupational time in active physical behaviors. Alternatively, the self-reported time devoted to Goldilocks-games could have been influenced by recall or reporting bias, affecting the accuracy of the time playing the games.

A key feature of Goldilocks Work is that it attempts to design work systems so that individual motivation to perform tasks in a particular way is less important. In this study, acting as role models for the children to increase physical activity through pedagogical games was highlighted as a key work task for childcare workers. However, since it is the childcare workers that are responsible for conducting the Goldilocks-games, it appears that they adapted the games to minimize their active role. Thus, despite our efforts to reduce individual motivation to be physical activity, it may still have been a factor that affected the extent to which some workers adapted the Goldilocks-games. For succeeding in changing behaviors at work, we recommend future research to develop implementation strategies among childcare workers that depend as little as possible on competence, preferences and individual motivation and to assess their effectiveness.

However, the satisfaction with the Goldilocks-games expressed by a majority of the childcare workers, their desire to continue with the games, and their recognition of the relevance of the games for other childcare institutions, combined with the commitment of the workers to play the games several times per week, suggest a general willingness to engage in the Goldilocks-games as intended. However, the considerable differences in needs of 3–6-year old children may have posed a challenge for the childcare workers in achieving high-intensity physical activity, despite their willingness to do so.

Enhancing the intervention to more effectively address the needs and preferences of childcare workers could be achieved by drawing insights from related studies within the childcare field, such as the TOY project, a participatory ergonomic intervention on physical exertion and musculoskeletal pain among childcare workers (30). In contrast to the present study, the TOY project used a participatory approach to identify and prioritize the contents of the intervention and its implementation, which eventually showed favorable outcomes among the childcare workers (30).

In contrast to the lack of intervention effect on high-intensity physical activity and active behaviors, the intervention resulted in an increase of 0.65 (95% CI 0.08–1.21) in perceived energy at work, measured on a scale from 0–10, in the intervention group compared to the control group, and a decrease of -0.32 (95% CI -0.54– -0.09) in need for recovery, measured on a scale from 1–5. The increase in perceived energy at work and the decreased need for recovery cannot be explained by changes in high-intensity physical activity. Other factors influencing perceived energy and need for recovery may be the positive effect of being outdoors, including reduced noise and a more pleasurable environment. Furthermore, participants’ belief that engaging in the Goldilocks-games would lead to health and fitness improvement may have contributed to the observed results, illustrating a possible placebo effect. It remains, however, uncertain whether the observed changes in perceived energy at work and need for recovery have any significant clinical or practical relevance.

We thus suggest that the lack of an intervention effect on occupational time in high-intensity physical activity was likely due to an implementation failure, ie, that the childcare workers did not sufficiently adhere to the Goldilocks-games protocol. During this study, it became clear that a deeper understanding of workers’ motivation for embracing an intervention is important. We therefore recommend future research acquires more knowledge on implementation strategies that can effectively increase occupational time in high-intensity physical activity among childcare workers.

### Strength and limitations

A strength of the present study was that consultants from WEC delivered the intervention. This indicates that the intervention is scalable without the involvement of the research group. Moreover, the consultants were not involved in the evaluation of the study, which contributes to maintaining objectivity and reducing bias. A second strength was that the study was conducted in real life working settings, making it easier to generalize the effects to similar workplaces. A final strength was the use of heart rate recordings to estimate the relative time in high-intensity physical activity for each individual childcare worker (31) and the use of validated accelerometer measurements to capture physical behaviors over multiple workdays (22).

A potential weakness of the study was the extensive exclusion of heart rate data due to a beat error ≥50%. A high beat error could possibly be explained by rapid body movements during the Goldilocks-games disturbing the signal. Therefore, occupational time in high-intensity physical activity may have been higher than reported in this study. Another limitation is the high loss to follow-up for primary and secondary outcomes, which is a common issue in intervention studies (32). Furthermore, the questions used to assess pain, energy at work and physical exhaustion need to be validated. Despite the challenging circumstances posed by the COVID-19 restrictions, >80% of the childcare workers reported that the restrictions had not or only to a low extent made it difficult to complete intervention activities. Thus, rather than illustrating a limitation, this indicates that the intervention is feasible to conduct even during challenging circumstances.

### Concluding remarks

In conclusion, this 8-week Goldilocks Work intervention was successfully delivered and well received among childcare workers, who reported to play Goldilocks-games for >110 minutes/week. However, the intervention had no effect on occupational time in high-intensity physical activity. The intervention group increased their energy at work and decreased their need for recovery, but showed no significant changes in the other health-related secondary outcomes. Further research on how to design and implement effective health-promoting work environments in childcare is needed.

## Supplementary material

Supplementary material
