# Supplementary material for: Effectiveness of a Goldilocks Work intervention in childcare workers – A cluster-randomized controlled trial
Source: Scand J Work Environ Health. 2024 Mar 27;50(3):197–207. doi: 10.5271/sjweh.4145 (PMC11057501; doi:10.5271/sjweh.4145)

Effectiveness of a Goldilocks Work intervention in childcare workers – A cluster-randomized controlled trial[1]

by Kathrine Greby Schmidt, MSc,[2] Anders Fritz Lerche, MSc, Marie Raunkjær Christensen, MSc, Charlotte Lund Rasmussen, PhD, Leon Straker, PhD, Svend Erik Mathiassen, PhD, Andreas Holtermann, PhD

1. Supplementary material
2. Correspondence to: Kathrine Greby Schmidt, The National Research Centre for the Working Environment, 2100 Copenhagen, Denmark. [E-mail: kgs@nfa.dk]

Appendix A. Description of intervention components that were modified due to requirements from the workplaces and to COVID-19 restrictions issued by the Danish Health Authority.

<table>
<tr><th>Modification</th><th>Activities planned according to the protocol</th><th>Activities completed</th><th>Reasons for the modification made</th></tr>
<tr><td>1. Length of intervention</td><td>10-weeks intervention period</td><td>8-weeks intervention period</td><td>The institutions had to remain closed for a month as part of the government's overall plan to reduce infection with COVID-19. Therefore, it was necessary to reduce the intervention period to ensure completion of data collection within the project period</td></tr>
<tr><td>2. Development of Goldilocks-games</td><td>During the first workshop, the childcare workers were planned to develop their own Goldilocks-games</td><td>In total, seven Goldilocks-games was developed. We collaborated with childcare workers to co-develop three Goldilocks-games. Later we developed additional four Goldilocks-games our self.</td><td rowspan="4">The childcare institutions had to adhere to several restrictions including physical distancing. Therefore, it was not possible to gather the childcare workers nor the children for a face-to-face workshop</td></tr>
<tr><td>3. Video material</td><td>It was planned to inform the childcare workers about the overall content of the Goldilocks Work Principle during a face-to-face workshop</td><td>A video with information of the overall content of the Goldilocks Work Principle and intervention content was sent individually to the childcare workers</td></tr>
<tr><td>4. Visit 1: Training and start-up of Goldilocks</td><td>The childcare workers were planned to develop their own Goldilocks-games during the first face-to-face workshop</td><td>Teaching childcare workers and children two Goldilocks-games</td></tr>
<tr><td>5. Visit 2: Further training and implementation</td><td>It was planned that during the second face-to-face workshop to the consultant</td><td>Teaching childcare workers and children two Goldilocks-games</td></tr>
</table>

6. Visit 3: Implementation and sustaining	and childcare workers would discuss the implementation of the Goldilocks-games, facilitate sustainability of well-functioning Goldilocks-games and modify the games if necessary	Teaching childcare workers and children three Goldilocks-games	
7. Collection of anthropometric and technical measurements	A representative from our research group measured the anthropometric and attached the monitors	A manual for self-conducting anthropometric measurements and self-application of the monitors was developed and used in some institutions	External visitors were not allowed to enter in all childcare institutions

Appendix B. Picture of how the poster and the colored paws were intended to be used. Each Goldilocks-game were giving its own paw color, the rows represent one intervention week and each color illustrates a specific Goldilocks-game. The children were encouraged to place a paw sticker on the poster every time they had played a Goldilocks-game.

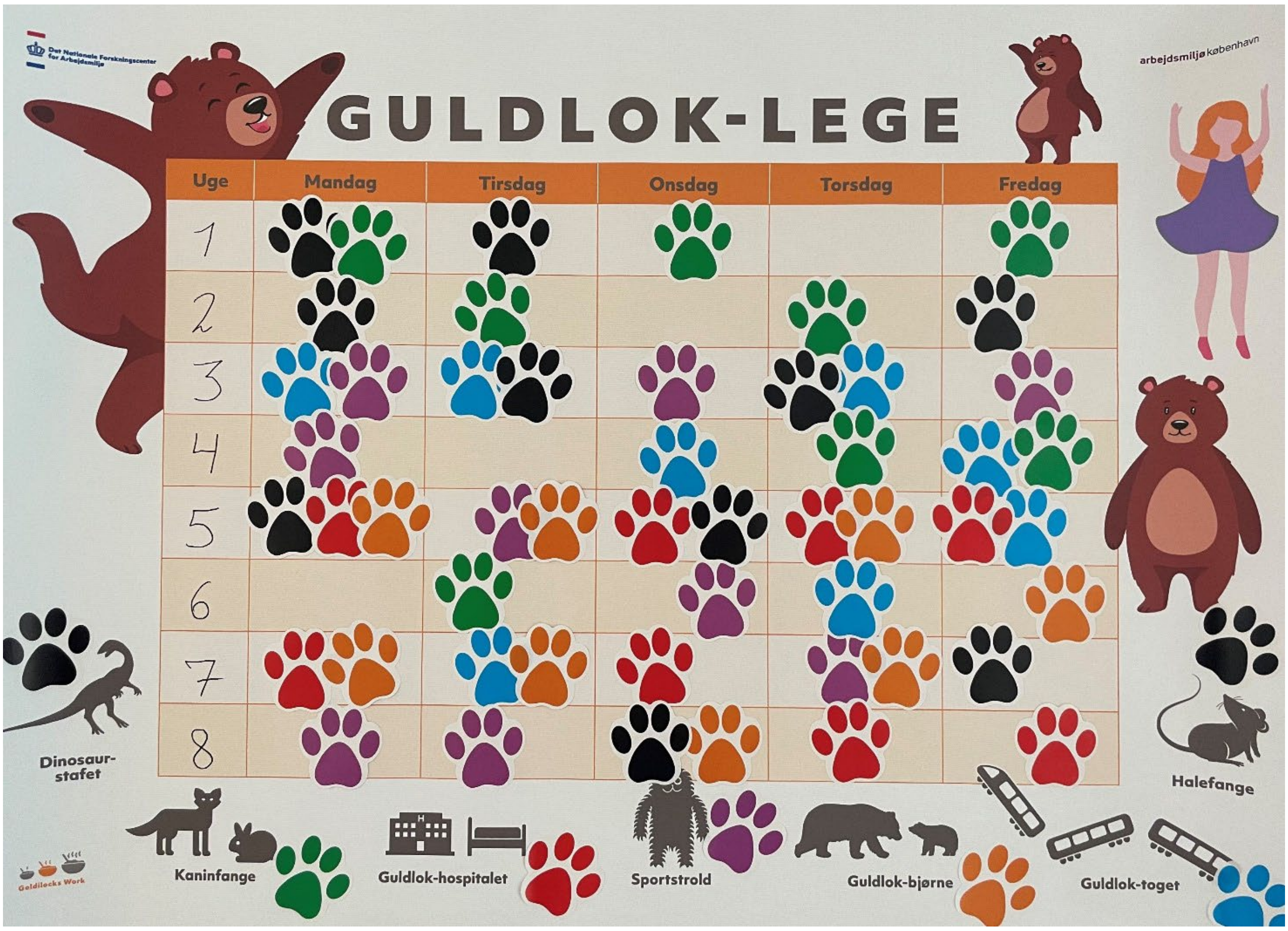

Supplement: Supplementary material [file SJWEH-50-197-S001.pdf]
